# Pre-therapeutic bone marrow-resident leukemic cells in acute myeloid leukemia exhibit a distinct dysregulated calcium signature and stem-like profile reflecting minimal residual disease precursors

**DOI:** 10.1186/s13046-025-03634-x

**Published:** 2026-01-09

**Authors:** Sofia Titah, Aurélie Guillemette, Clara Lewuillon, Faruk Azam Shaik, Céline Berthon, Laure Goursaud, Meryem Tardivel, Antonino Bongiovanni, Paul Chauvet, Nathalie Jouy, Pauline Peyrouze, Meyling Cheok, Carine Brinster, Salomon Manier, Mehmet Çagatay Tarhan, Loïc Lemonnier, Bruno Quesnel, Yasmine Touil

**Affiliations:** 1 PERSTIM lab, CRCLille, Inserm U1366, CNRS UMR9020, ONCOLille, University of Lille, Institut Pasteur Lille (IPL), CHU Lille, Lille, F-59000 France; 2 Univ. Lille, CNRS, Inserm, CHU Lille, Institut Pasteur de Lille, UMR9020- U1366-CRCLILLE-Cancer Research Center of Lille, Lille, 59000 France; 3 Univ. Lille, CNRS, Centrale Lille, Junia, Univ. Polytechnique Hauts-de-France, UMR 8520 -IEMN -Institut d’Electronique de Microélectronique Et de Nanotechnologie, Lille, 59000 France; 4 Univ. Lille, CNRS, Inserm, CHU Lille, Institut Pasteur de Lille, US 41 - UAR 2014 - PLBS, Lille, 59000 France

**Keywords:** AML, MRD, Bone marrow PDX, Calcium signaling, Stem cells, ORAI channels

## Abstract

**Background:**

Acute myeloid leukemia (AML) remains a high-risk hematologic malignancy due to frequent relapse and therapeutic resistance. Although induction therapy can achieve cytological remission, a fraction of leukemic cells (minimal residual disease, MRD) persists within the protective bone marrow (BM) microenvironment. MRD is heterogeneous and may include subclones with intrinsic survival features present before therapy. Among these, rare BM-resident leukemic cells (BMresLC) may represent pre-adapted precursors of MRD, maintained in a low-proliferative (Ki67^low^) or quiescent state. We previously showed that calcium signaling through ORAI1-dependent store-operated calcium entry (SOCE) contributes not only to AML stemness and drug resistance but also to the regulation of the G0-G1 cell-cycle transition and the emergence of slow-cycling leukemic cells. With this study, we have characterized the stemness and calcium signature of BMresLC before any therapeutic intervention. Our results, beyond further characterizing a population of cells rarely studied, could thus pave the way to new therapeutic opportunities combining current treatments with the targeting of relevant pathways highlighted by our work.

**Methods:**

A patient-derived xenograft (PDX) model in NSG (NOD/SCID/IL2Rγ^null^) mice was used to localize, isolate, and characterize human BMresLC. Whole-bone clearing and 3D-Imaris imaging enabled spatial localization of rare leukemic cells. Flow cytometry and qPCR assessed cell-cycle status, immunophenotype (CD34, CD38, TIM-3, PD-L1, Ki67), stemness, and calcium-signaling components (*ORAI1-3*, *STIM1-2*, *NFATc1-4*). SOCE was measured using Indo-1 assays. Comparative analyses were performed against diagnostic AML cells, public MRD RNA-seq datasets, and prognosis-stratified patient cohorts.

**Results:**

BMresLC displayed an immune-evasive immunophenotype and contained a small fraction of Ki67^neg^ quiescent cells, but were not enriched in fully quiescent cells. Instead, they predominantly exhibited a Ki67^low^ slow-cycling profile, consistent with a low-proliferative persistent state. Transcriptional analysis revealed overexpression of stemness-associated genes and selective downregulation of calcium-signaling components *ORAI1, ORAI2, STIM2*, and *NFATc1/c4*, consistent with a SOCE-suppressed calcium signature. Functional assays confirmed reduced calcium influx. Compared with post-therapy MRD datasets, BMresLC showed some stemness and immune-evasion traits but displayed a distinct pre-therapeutic calcium signature, suggesting that it represents an early, persistent state preceding full MRD remodeling. Prognostic subgroup analysis further showed that BMresLC calcium and stemness profiles partially recapitulate features of adverse-risk AML, including differences in CD34, CD38, PD-L1, *MMRN1*, *LAPTM4B*, *NFATc2*, and *STIM2* expression.

**Conclusions:**

Our findings identify a distinctive calcium- and stemness-based signature in BMresLC, representing a pre-MRD survival state characterized by slow cycling rather than enrichment in strict quiescence. This pre-therapeutic signature may contribute to MRD establishment and relapse risk in AML.

**Supplementary Information:**

The online version contains supplementary material available at 10.1186/s13046-025-03634-x.

## Introduction

Acute myeloid leukemia (AML) is a genetically and phenotypically heterogeneous hematologic malignancy characterized by the clonal expansion of immature myeloid blasts in the bone marrow (BM) and peripheral blood [1, 2]. Despite initial remission rates exceeding 70% with intensive induction chemotherapy, the majority of patients relapse due to the persistence of therapy-resistant leukemic cells, often referred to as minimal residual disease (MRD) [1, 3]. These residual cells are not only clinically occult but also molecularly distinct, often harboring features that allow immune evasion, chemoresistance, and metabolic adaptation [4, 5]. While much attention has been devoted to characterizing MRD after chemotherapy, far less is known about the treatment-naïve leukemic cells that persist deep within the BM niche before any therapeutic pressure is applied. In AML, leukemic stem cells (LSCs) have long been proposed to contribute to relapse by withstanding chemotherapy [6]. The relationship between pre-treatment LSC-like cells residing in the BM and post-treatment MRD remains however insufficiently defined. Current studies indicate that MRD after therapy can contain deeply quiescent, metabolically adapted, or immune-evading subpopulations, yet the precise nature and origin of these MRD-driving cells remain controversial across AML cohorts [7–9].

This raises an important question: do pre-therapeutic BM-resident leukemic cells represent an early, niche-adapted survival state that precedes MRD, or do MRD populations arise through additional treatment-induced selection and reprogramming? Few studies have directly examined the phenotype and functional properties of leukemic cells that persist before treatment, despite evidence that the BM niche can shelter slow-cycling or stem-like clones with intrinsic survival circuits [7, 10]. Understanding whether such pre-treatment BM-resident leukemic cells exist, what their biological features are, and how they differ from post-treatment MRD is essential for clarifying the continuum of AML persistence and identifying opportunities for early therapeutic intervention.

Calcium signaling has recently emerged as a critical regulator of stem cell fate and leukemic persistence [11]. Store-operated calcium entry (SOCE), mediated by the ORAI-STIM complex, represents the main calcium entry pathway in non-excitable cells [12, 13]. SOCE modulates not only T-cell activation but also survival and proliferation of LSC [14, 15]. Moreover, Nuclear Factor of Activated T cells (NFAT) transcription factors, activated downstream of calcium influx [16], have been implicated in drug tolerance and immune suppression in various hematological malignancies [17]. In the hematopoietic system, mitochondrial calcium uptake regulates stem cell division and lineage priming, further highlighting calcium’s role in cell fate [18–20]. We previously demonstrated that altered ORAI1-dependent SOCE signaling impacts AML cell-cycle progression, promotes stem-like transcriptional programs, and enhances drug tolerance, thereby linking calcium-channel dysregulation to key biological hallmarks associated with MRD formation and relapse [21]. Therefore, investigating the calcium signature in rare BM-resident leukemic cells termed BMresLC naïve of treatment may help better understand MRD cell “initial” intrinsic characteristics.

In this study, we utilized a patient-derived xenograft (PDX) model in NOD/SCID/IL2Rγc^null^ (NSG) mice to isolate and functionally characterize rare BMresLC. Advanced imaging and flow cytometry profiling enabled us to define their spatial localization, cell cycle status, and immunophenotype. We compared BMresLC samples with matched leukemic cells from adult de novo AML patients at diagnosis and at MRD, and further integrated large public transcriptomic datasets to evaluate immune evasion, stemness, and calcium-related gene programs stratified by clinical prognosis. Our findings unveil a distinct calcium-NFAT signature in BMresLC, different from both naïve diagnostic blasts and post-treatment MRD cells, indicating early, intrinsic survival programs linked to stemness, cell cycle, immune evasion, and calcium signaling. These results provide new insights into the biological identity of residual leukemia and identify calcium signaling pathways as early and potentially targetable mediators of relapse in AML.

## Results

### Detection of a rare BMresLC compartment composed of minor quiescent cells and a dominant slow-cycling population in AML xenografts

**Fig. 1:**
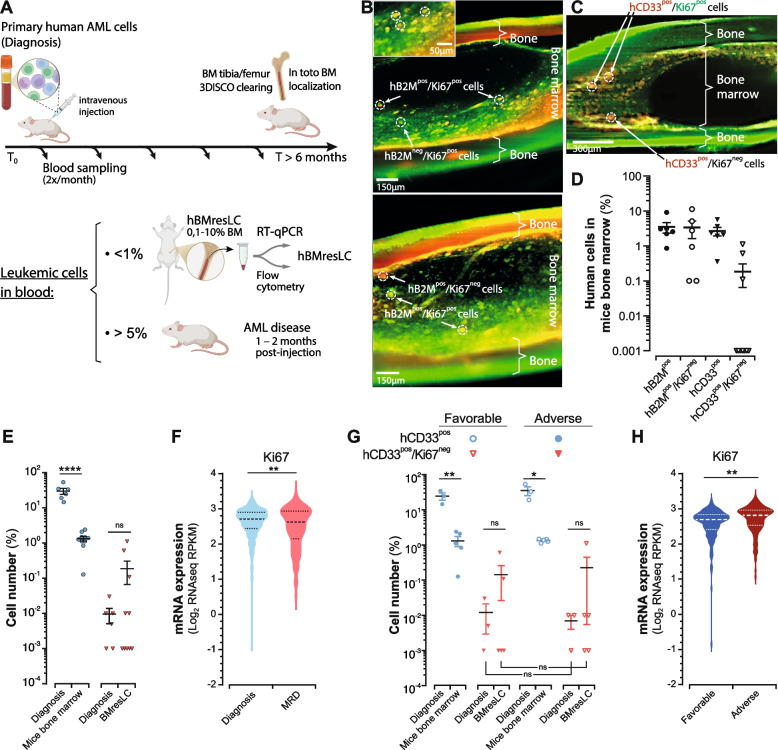
Identification and characterization of rare human BM-resident leukemic cells (BMresLC) in a xenograft model. **A** Experimental workflow: primary human AML cells were intravenously injected into immunodeficient mice. Peripheral blood was sampled twice monthly and analyzed by flow cytometry for human CD45^pos^ cells. At endpoint (≥ 180 days), mice were sacrificed, and BM was collected for RT-qPCR, flow cytometry, and 3D imaging after 3DISCO tissue clearing. **B** Representative 3D-IMARIS reconstructions of cleared tibial/femoral BM, stained for β2-microglobulin (human MHC class I subunit, red) and Ki67 (proliferation marker, green), showing human B2M^pos^ and Ki67^pos^ cells or their negative counterparts. Images illustrate the localization and proliferative state of hBMresLC (e.g., B2M^pos^/Ki67^pos^ and B2M^pos^/Ki67^neg^ subpopulations) in different AML samples. Scale bar:150 µm. **C** Immunofluorescence staining of 3D-cleared BM tissue with anti-human CD33-PeCy7 (myeloid marker, red) and anti-Ki67-FITC (proliferation marker, green) showing spatial localization of hCD33^pos^/Ki67^pos^ and hCD33^pos^/Ki67^neg^ cell subpopulations. Scale bar: 300 µm. **D** Flow cytometry-based quantification of human leukemic cells in the BM mouse model, based on B2M, CD33, and Ki67 expression level (Ki67^neg^: quiescent cells). **E** Flow cytometry-based quantification of human CD33^pos^ cells (blue) in diagnosis and mice BM, and the proportion of CD33^pos^ cells lacking Ki67 expression (CD33^pos^/Ki67^neg^ quiescent cells) (red) in diagnosis and BMresLC samples. Statistical analysis was performed using Student’s *t* test (**** *p <* 0.0001, non-significant (ns)). **F** Violin plots illustrating mRNA expression levels of *Ki67* across distinct clinical subgroups. RNA-seq data were generated from samples at AML diagnosis (*n =* 445) and MRD (*n =* 130) [9, 23, 24]. RNA-Seq data were obtained from public domain AML datasets (https://www.cbioportal.org) and extracted as described in [25]. Each violin represents the distribution of log₂ RPKM expression values. The central dotted marker (larger dot) indicates the median, while the upper and lower dotted markers (smaller dots) represent the interquartile range (IQR). Statistical significance between groups was assessed using the Student *t*-test. (**p < 0.01). **G** Quantification according to patient prognosis at diagnosis (adverse *vs* favorable) and among BMresLC from BM mouse model according to the AML prognosis (adverse *vs* favorable). Statistical analysis was performed using Student’s *t* test (** *p <* 0.01, non-significant (ns)). **H** Violin plots showing *Ki67* mRNA expression levels in patient samples stratified according to adverse versus favorable prognosis (public datasets [23], cBioportal). Each violin represents the distribution of log₂ RPKM expression values; The central dotted marker (larger dot) indicates the median, while the upper and lower dotted markers (smaller dots) represent the interquartile range (IQR), and the shape reflects data density. Statistical significance between groups was assessed using the Student *t*-test (**p < 0.01, non-significant (ns))

To investigate the mechanisms underlying leukemic persistence and MRD, we utilized a xenotransplantation model wherein primary human AML blasts were intravenously injected into immunodeficient NSG mice (Fig. 1A). Approximately 40–60% of recipient animals developed systemic leukemia within 1–2 months post-engraftment. In contrast, the remaining mice demonstrated a spontaneous decline of circulating human CD45^pos^ cells to below-detection thresholds (< 0.01%) or maintained low-level persistence (0.01–1%) over long-term follow-up (≥ 180 days). Despite the absence of detectable circulating AML cells, post-mortem analysis of femoral and tibial BM revealed a persistent population of human leukemic cells, representing 0.1–10% of total BM cellularity. These rare cells were identified using human-specific markers, β2-microglobulin (hB2M) and hCD33, which reliably discriminate human AML blasts from murine hematopoietic cells in PDX systems [22]. Their presence was confirmed by 3D imaging of optically cleared bone tissue, in which rare hB2M^pos^ and hCD33^pos^ human leukemic cells were visualized within discrete BM niches corresponding to canonical BM microenvironments, notably the peri-endosteal and perivascular regions, both of which are well-described reservoirs for LSC and therapy-persistent MRD-like cells (Fig. 1B, C). Consistently, RT-qPCR analysis detected human *B2M* and *CD33* transcripts in the same samples, confirming the human leukemic origin of these residual cells. We next quantified this BM-resident human leukemic compartment by flow cytometry (Fig. 1D). Figure 1D shows the proportion of hB2M^pos^ and hCD33^pos^ cells among total murine BM mononuclear cells. These measurements confirm that BMresLC represent a small but consistent human leukemic population persisting in the BM of xenografted mice. The variability in the proportion of BMresLC was observed across individual xenografted mice, even when different animals were transplanted with cells from the same AML patient sample (Fig S1A). This variability likely reflects differences in BMresLC dissemination and niche residency among mice. Nonetheless, despite this inter-mouse variation, the overall frequency of BMresLC remains consistently low across all xenografts, supporting the robustness of this residual BM-resident leukemic compartment.

Combination with Ki67 staining allowed us to assess the cycling (Ki67^pos^) and the quiescent (Ki67^neg^) state of the BMresLC. Immunofluorescence imaging and flow cytometry revealed clear proliferative heterogeneity, with BMresLC comprising both Ki67^pos^ cycling cells and a minor fraction of Ki67^neg^ quiescent cells (Fig. 1B, C, D). We next compared the hCD33^pos^ cell compartment between the mouse BM environment, including the BMresLC, and diagnostic AML samples (Fig. S1B). We assessed their proportion in quiescent cells by Ki67 expression analysis (Fig. 1E). As expected, hCD33^pos^ cells represented a small fraction of total BM mouse mononuclear cells (mean 1.48%) compared with 56.8% at diagnosis. Importantly, both hCD33^pos^/Ki67^neg^ quiescent leukemic cells were detected in both conditions, and no significant difference in the proportion of Ki67^neg^ cells was observed between BMresLC and diagnostic blasts, confirming that BMresLC are not enriched in strictly quiescent cells. Interestingly, BMresLC were enriched in slow-cycling cells, as revealed by a significant increase in Ki67^low^ cells compared to the diagnosis stage (Fig. S1D). Overall, these data indicate that, although the BM-resident compartment is not enriched in fully quiescent populations, it contains rare quiescent cells integrated within a predominantly slow-cycling (Ki67^low^) landscape, consistent with a persistent but not fully dormant leukemic reservoir.

We then analyzed publicly available RNA-seq datasets comparing post-treatment MRD cells and diagnostic AML samples, using adult de novo AML cohorts published by Tyner et al. [23], Bottomly et al*.* [24], and Kim et al*.* [9], all accessible through cBioPortal. These datasets include detailed clinical information, allowing us to assess differences in proliferation status, including Ki67 expression, and to relate these transcriptional programs to disease stage progression (diagnosis and MRD) and clinical prognosis. MRD cells exhibited a significant reduction in *Ki67* mRNA (*p <* 0.01), consistent with a more deeply quiescent post-treatment state and/or a slow cycling phenotype (Fig. 1F).

Finally, when stratifying by AML disease prognosis (Table S1), BMresLC from adverse-risk and favorable-risk patients did not differ significantly in their proportion of quiescent hCD33^pos^/Ki67^neg^ cells (Fig. 1G). By contrast, in large patient datasets, *Ki67* mRNA was significantly higher at diagnosis in adverse-risk AML (Fig. 1H), indicating that *Ki67* transcript abundance reflects disease biology but cannot be used to discriminate quiescent from slow-cycling cells. Consistent with this limitation, RNA-seq analyses cannot distinguish between truly quiescent (Ki67^neg^) and low-proliferative (Ki67^low^) states, as both can result in reduced *Ki67* mRNA levels. Altogether, our findings indicate that BMresLC persist in a low-proliferative (Ki67^low^) state with only a minor Ki67^neg^ fraction, and that this phenotype is independent of AML prognostic risk. In MRD RNA-seq datasets, *Ki67* mRNA is globally reduced after therapy; however, this decrease cannot be interpreted as definitive evidence of quiescence and may equally reflect a shift toward a deeper low-proliferative or slow-cycling state. Nevertheless, multiple studies [26–28] have reported that post-chemotherapy MRD compartments frequently contain more deeply quiescent LSC-like cells, including G0-enriched subsets. Although reduced *Ki67* mRNA in MRD RNA-seq datasets cannot conclusively distinguish true quiescence from slow cycling, this consistent decrease aligns with the post-treatment quiescence signatures described in the literature. Thus, while BMresLC reflect a pre-MRD, slow-cycling population conditioned by the BM niche before therapy, MRD cells may correspond to a post-therapy, more profoundly quiescent state, consolidated by chemotherapeutic selection. This distinction reinforces the idea that MRD quiescence emerges largely after treatment, whereas BMresLC could represent an earlier, transcriptionally primed but less deeply quiescent reservoir.

### BMresLC partially retain a stem-like, drug-resistant, and immunoevasion phenotype of MRD cells

**Fig. 2:**
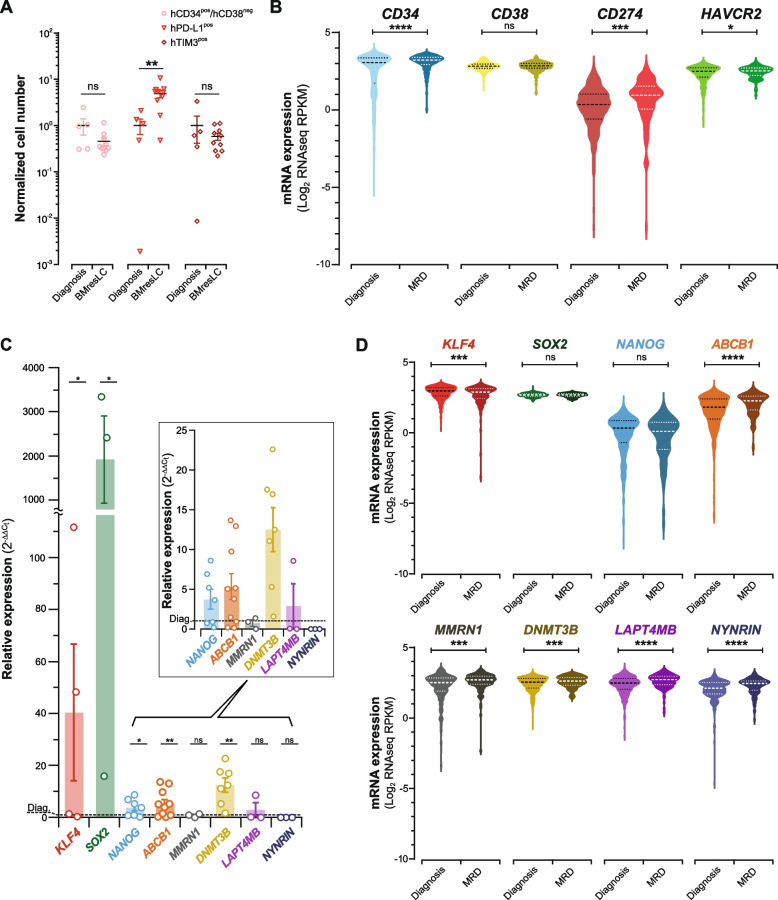
BMresLC partially retain phenotypic and transcriptional features related to immunoevasiveness, stemness, and drug-resistance related genes of MRD cells. **A** Flow cytometry-based quantification of human CD34^pos^/CD38^neg^ cells (pink dot), PD-L1^pos^ cells (red triangle), and TIM-3^pos^ cells (dark red square) in patient samples at diagnosis and among hBMresLC from xenografted mouse BM. Values are normalized to the diagnostic stage. Statistical significance between groups was assessed using the Student’s *t*-test. (**p < 0.01). **B** Violin plots illustrating mRNA expression levels of stem cell markers *CD34* and *TIM-3*, differentiation marker *CD38*, and an immunoevasion marker *CD274* (PD-L1) at diagnosis (*n =* 428) and MRD stage (*n =* 128) (public datasets [9, 23, 24], cbioportal). Each violin represents the distribution of log₂ RPKM expression values; The central dotted marker (larger dot) indicates the median, while the upper and lower dotted markers (smaller dots) represent the interquartile range (IQR), and the violin shape reflects data density. Significant upregulation of *CD34*, *CD274,* and *HAVCR2* was observed in MRD cells (**** *p <* 0.0001, *p < 0.05; **p < 0.01, non-significant (ns)), consistent with a more primitive and immune-evasive phenotype. Statistical significance between groups was assessed using the Student’s *t*-test (*p < 0.05, ***p < 0.001, ****p < 0,0001, non-significant (ns)). **C** Relative *KLF4, SOX2, NANOG, ABCB1, MMRN1, DNMT3B, LAPT4MB, NYNRIN* gene expression in BMresLC compared to AML cells at diagnosis stage (dashed line) and determined by RT-qPCR. *KLF4, SOX2, NANOG, ABCB1, MMRN1, DNMT3B, LAPT4MB, NYNRIN* relative gene expression was calculated by the 2^−ΔΔCt^ method. Statistical significance was assessed using the Student’s *t*-test (*p < 0.05, **p < 0,01, non-significant (ns)). **D** Violin plots illustrating mRNA expression levels of *KLF4, SOX2, NANOG, ABCB1, MMRN1, DNMT3B, LAPT4MB, NYNRIN* at diagnosis (*n =* 447) and MRD stage (*n =* 134) (public dataset [9, 23, 24], cBioportal). Each violin represents the distribution of log₂ RPKM expression values; The central dotted marker (larger dot) indicates the median, while the upper and lower dotted markers (smaller dots) represent the interquartile range (IQR), and the violin shape reflects data density. Statistical significance between groups was assessed using the Student’s *t*-test (***p < 0.001, ****p < 0,0001, non-significant (ns))

To further characterize the phenotype of BMresLC, we conducted multiparametric flow cytometry and transcriptomic profiling, comparing them with matched diagnostic AML samples. BMresLC contained a detectable CD34^pos^/CD38^neg^ and TIM-3^pos^ fraction, indicating that an immature, LSC-like subpopulation is preserved within the residual BM compartment, even though no enrichment of CD34^pos^/CD38^neg^ surface marker expression was observed compared with diagnostic AML samples (Fig. 2A, Fig. S2A, B). Importantly, surface expression of the immune checkpoint ligand PD-L1 (*CD274*) was significantly elevated in BMresLC compared to their diagnostic counterparts (*p <* 0.01; Fig. 2A, Fig. S2A, B). To assess similarity to MRD post-chemotherapy cells, we analyzed public RNA-seq datasets [9, 23, 24] comparing diagnostic *versus* MRD. MRD cells exhibited increased expression of *CD34* (*p <* 0.0001), alongside elevated *CD274* (PD-L1) and *HAVCR2* (TIM-3) (*p <* 0.001 and *p <* 0.05, respectively), consistent with a stem-like, immune-evasive profile (Fig. 2B). These features partially overlap with BMresLC, indicating that this residual pre-treatment population may represent a transitional state with molecular predisposition toward chemoresistance and immune escape. To elucidate molecular pathways underpinning treatment resistance, we analyzed the expression of pluripotency and drug resistance-associated genes in BMresLC and compared them with matched diagnostic AML samples. Quantitative PCR revealed that BMresLC exhibited significantly elevated expression of *KLF4, SOX2, NANOG, DNMT3B,* and *ABCB1* (Fig. 2C). This pattern suggests a reprogrammed, stem-like transcriptional state with multidrug resistance potential. Comparison with public RNA-seq data [9, 23, 24] from post-chemotherapy MRD AML samples revealed partial overlap: MRD cells showed upregulation of *ABCB1, MMRN1, DNMT3B, LAPTM4B,* and *NYNRIN*, while pluripotency genes such as *SOX2* and *NANOG* remained unchanged except for *KLF4* (Fig. 2D). Interestingly, genes from the well-established LSC17 signature [28], including *MMRN1*, *LAPTM4B,* and *NYNRIN*, were more prominent in MRD than BMresLC, indicating that distinct transcriptional programs govern pre-therapy persistence versus post-treatment resistance.

### BMresLC display a stem-like and immune checkpoint-enriched phenotype that mirrors the immunophenotypic profile of diagnostic-stage AML cells from high-risk patient profiles

**Fig. 3:**
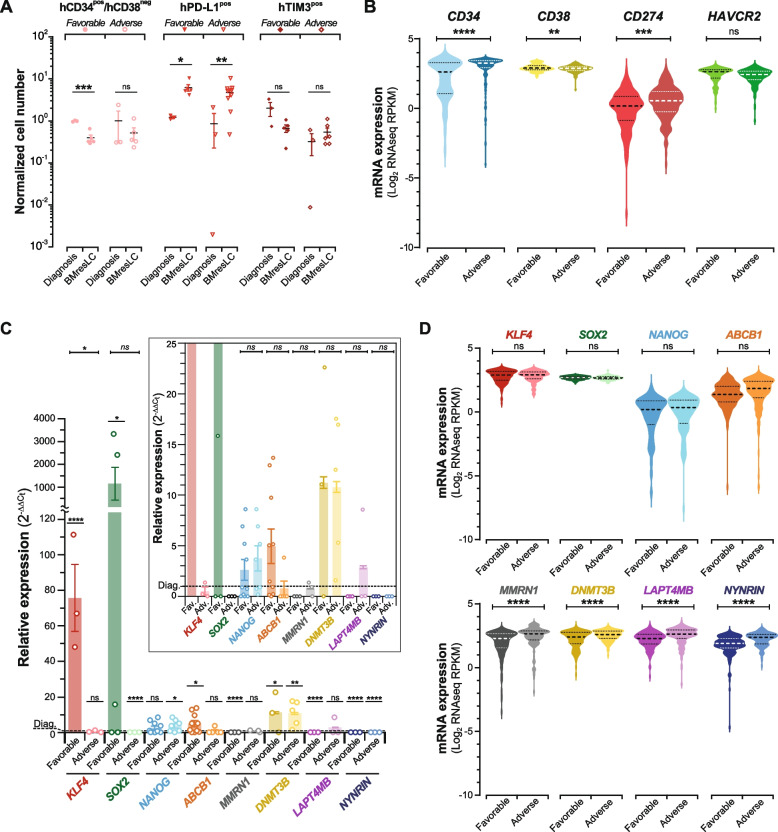
BMresLC cells from patients with an adverse prognosis display similarity to the stemness program of high-risk AML cells from the diagnosis stage. **A** Flow cytometry–based quantification of the percentage of human CD34^pos^/CD38^neg^ cells (pink), PD-L1^pos^ cells (red), and TIM-3^pos^ cells (dark red) at diagnosis according to the AML prognosis (adverse-risk *vs* favorable-risk) and among hBMresLC from the BM mouse model according to the AML prognosis (adverse *vs* favorable). Values are normalized to the diagnosis stage. **B** Violin plots showing mRNA expression levels of *CD34*, *CD38*, *CD274* (PD-L1), and *HAVCR2 (*TIM-3*)* across adverse and favorable prognosis groups (public dataset [9, 23, 24], cbioportal). Each violin represents the distribution of log₂ RPKM expression values; The central dotted marker (larger dot) indicates the median, while the upper and lower dotted markers (smaller dots) represent the interquartile range (IQR), and the violin shape reflects data density. Statistical significance between groups was assessed using the Student’s *t*-test (**p < 0.01, ***p < 0.001, **** *p <* 0.0001, non-significant (ns)). Poor-outcome AML cells exhibit a gene expression signature marked by high *CD34*, *CD274,* and low *CD38*, closely resembling the phenotype landscape of poor-outcome BMresLC. **C** Relative *KLF4, SOX2, NANOG, ABCB1, MMRN1, DNMT3B, LAPT4MB, NYNRIN* gene expression in BMresLC compared to AML cells at diagnosis stage and according to the clinical outcome (adverse *vs* favorable) and determined by RT-qPCR. *KLF4, SOX2, NANOG, ABCB1, MMRN1, DNMT3B, LAPT4MB, NYNRIN* relative gene expression was calculated by the 2^−ΔΔCt^ method. Statistical analysis was performed using Student’s *t* test (* *p <* 0.05, ** *p <* 0.01, *** *p <* 0.001, ****p < 0,0001, non-significant (ns)). **D** Violin plots illustrating mRNA expression levels of *KLF4, SOX2, NANOG, ABCB1, MMRN1, DNMT3B, LAPT4MB,* and *NYNRIN* in AML cells from patients with adverse (*n =* 172) and favorable prognosis (*n =* 158) (public datasets [9, 23, 24], cbioportal). Each violin represents the distribution of log₂ RPKM expression values; The central dotted marker (larger dot) indicates the median, while the upper and lower dotted markers (smaller dots) represent the interquartile range (IQR), and the violin shape reflects data density. Statistical significance between groups was assessed using the Student’s *t*-test (****p < 0,0001). Marks were added to distinguish between comparison to diagnosis or between adverse-risk and favorable-risk conditions

To determine the clinical relevance of the BMresLC profile, we stratified their phenotypes and transcriptional patterns according to AML prognosis. BMresLC from adverse-risk AML maintained high expression of CD34^pos^/CD38^neg^ and TIM-3^pos^ markers compared to favorable-risk profiles, which exhibited a notable reduction in stem-like subsets (Fig. 3A). PD-L1^pos^ cells remained elevated in BMresLC regardless of prognostic group. Transcriptomic analysis of AML patient samples [9, 23, 24] stratified by clinical outcome revealed that patients with adverse risk had significantly increased gene expression of *CD34*, *CD274,* and lower *CD38* levels (Fig. 3B), consistent with the BMresLC phenotype. In contrast, favorable-risk AML cells demonstrated reduced expression of *CD34* and *CD274,* and higher expression of *CD38*, indicative of a more differentiated state and less immunoevasion features. These data position BMresLC, particularly from adverse-risk cases, as faithful preclinical surrogates of relapse-prone MRD. To determine whether these molecular differences were associated with clinical risk, we stratified BMresLC gene expression according to prognostic subgroups. BMresLC from favorable-risk AML showed increased gene expression of *KLF4, SOX2,* and *ABCB1*, but decreased gene expression of *LAPTM4B* (Fig. 3C). In contrast, adverse-risk BMresLCs showed lower gene expression of *KLF4*, *SOX2*, but maintained high *NANOG* and *DNMT3B* levels and low *NYNRIN* levels. These profiles partially mirror RNA-seq patterns from adverse-risk AML patients, who showed enrichment in stemness-associated genes such as *MMRN1*, *DNMT3B*, and *LAPTM4B* (Fig. 3D). These findings suggest that BMresLC from adverse-risk AML cases retain a stem-like transcriptional and immunophenotypic profile closely aligned with high-risk diagnostic blasts and relapse-prone MRD clones. In contrast, BMresLC from favorable-risk patients exhibit a more reprogrammed, pluripotency-enriched signature, potentially reflecting a distinct path of residual cell evolution with reduced relapse potential.

### BMresLC downregulate calcium signaling components in contrast with MRD cells

**Fig. 4:**
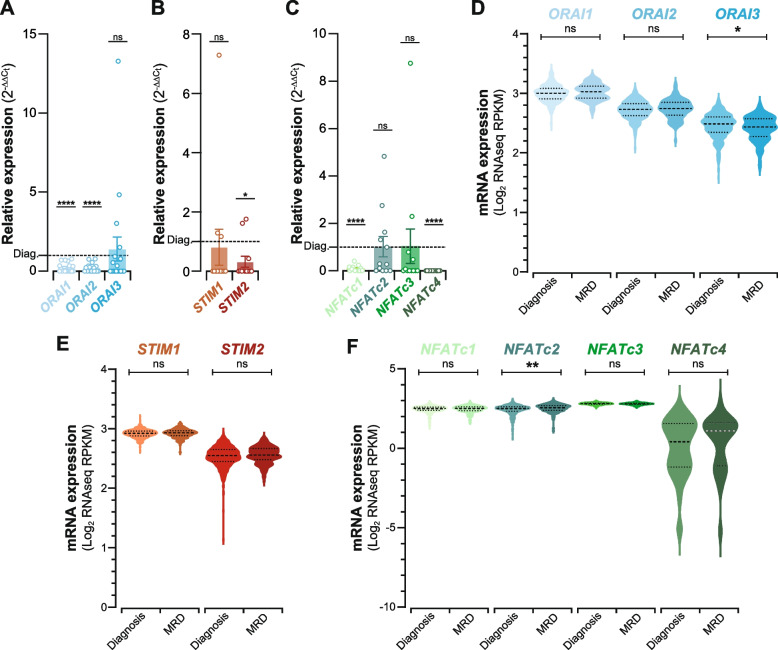
BMresLC downregulate calcium signaling components in contrast with MRD cells. **A** Relative *ORAI1*, *ORAI2*, *ORAI3* gene expression in BMresLC compared to AML cells at diagnosis stage determined by RT-qPCR. *ORAI1*, *ORAI2*, and *ORAI3* relative gene expression was calculated by the 2^−ΔΔCt^ method. Statistical significance was performed using Student’s *t* test (**** *p <* 0.0001). **B** Relative *STIM1*, *STIM2* gene expression in BMresLC compared to AML cells at the diagnosis stage was determined by RT-qPCR. *STIM1*, *STIM2* relative gene expression were calculated by the 2^−ΔΔCt^ method. Statistical significance was performed using Student’s *t* test (* *p <* 0.05). **C** Relative *NFATc1*, *NFATc2*, *NFATc3*, and *NFATc4* gene expression in BMresLC compared to AML cells at the diagnosis stage, determined by RT-qPCR. *NFATc1*, *NFATc2*, *NFATc3*, and *NFATc4* relative gene expression was calculated by the 2^−ΔΔCt^ method. Statistical significance was performed using Student’s *t* test (* *p <* 0.05, **** *p <* 0.0001). **D** Violin plots illustrating mRNA expression levels of *ORAI1*, *ORAI2*, and *ORAI3* in AML cells at diagnosis (*n =* 450) and MRD stage (*n =* 131) (public datasets, [9, 23, 24] cBioportal). Each violin represents the distribution of log₂ RPKM expression values; The central dotted marker (larger dot) indicates the median, while the upper and lower dotted markers (smaller dots) represent the interquartile range (IQR), and the violin shape reflects data density. Statistical significance was performed using Student’s *t-test* (* *p <* 0.05). **E** Violin plots illustrating mRNA expression levels of *STIM1*, *STIM2* in AML cells at diagnosis (*n =* 450) and MRD/Residual stage (*n =* 131) (public datasets, [9, 23, 24] cBioportal). Each violin represents the distribution of log₂ RPKM expression values; The central dotted marker (larger dot) indicates the median, while the upper and lower dotted markers (smaller dots) represent the interquartile range (IQR), and the violin shape reflects data density. **F** Violin plots illustrating mRNA expression levels of *NFATc1, NFATc2, NFATc3*, and *NFATc4* in AML cells at diagnosis (*n =* 450) and MRD stage (*n =* 131) (public datasets, [9, 23, 24] cBioportal). Each violin represents the distribution of log₂ RPKM expression values; The central dotted marker (larger dot) indicates the median, while the upper and lower dotted markers (smaller dots) represent the interquartile range (IQR), and the violin shape reflects data density. Statistical significance between groups was assessed using the Student’s *t* test (**p < 0.01)

To explore the role of calcium signaling in residual leukemia biology, we next analyzed the expression of key components in the SOCE pathway. qPCR analysis revealed that BMresLC significantly downregulated *ORAI1* and *ORAI2* expression, with no change in *ORAI3* levels compared to diagnosis (Fig. 4A). This was accompanied by reduced expression of the calcium sensor *STIM2* (Fig. 4B) and decreased transcription of *NFATc1* and *NFATc4*, but not *NFATc2* or *NFATc3* (Fig. 4C). In contrast to BMresLC, RNA-seq public datasets [9, 23, 24] from post-treatment MRD samples revealed a distinct remodeling of the Ca^2^⁺-signaling pathway. The expression of the main SOCE components *ORAI1* and *ORAI2*, as well as *ORAI3*, was assessed first (Fig. 4D). While *ORAI1* and *ORAI2* remained stable, *ORAI3* showed a slight but significant decrease (*p <* 0.05) in MRD samples. Analysis of the endoplasmic reticulum (ER) Ca^2^⁺ sensors *STIM1* and *STIM2* (Fig. 4E) showed no significant change in MRD compared with diagnostic AML. In contrast, MRD samples exhibited a selective upregulation of *NFATc2* (Fig. 4F), whereas *NFATc1*, *NFATc3* and *NFATc4* remained unchanged. This isoform-specific activation of *NFATc2* could highlight a post-treatment shift toward a calcineurin/NFATc2-driven persistent program. These findings suggest that BMresLC exhibit a “calcium signaling-hypoactive” phenotype, whereas MRD cells may reactivate calcium/NFAT signaling post-treatment.

### Calcium signaling gene expression stratifies AML cells and BMresLC by prognosis

**Fig. 5:**
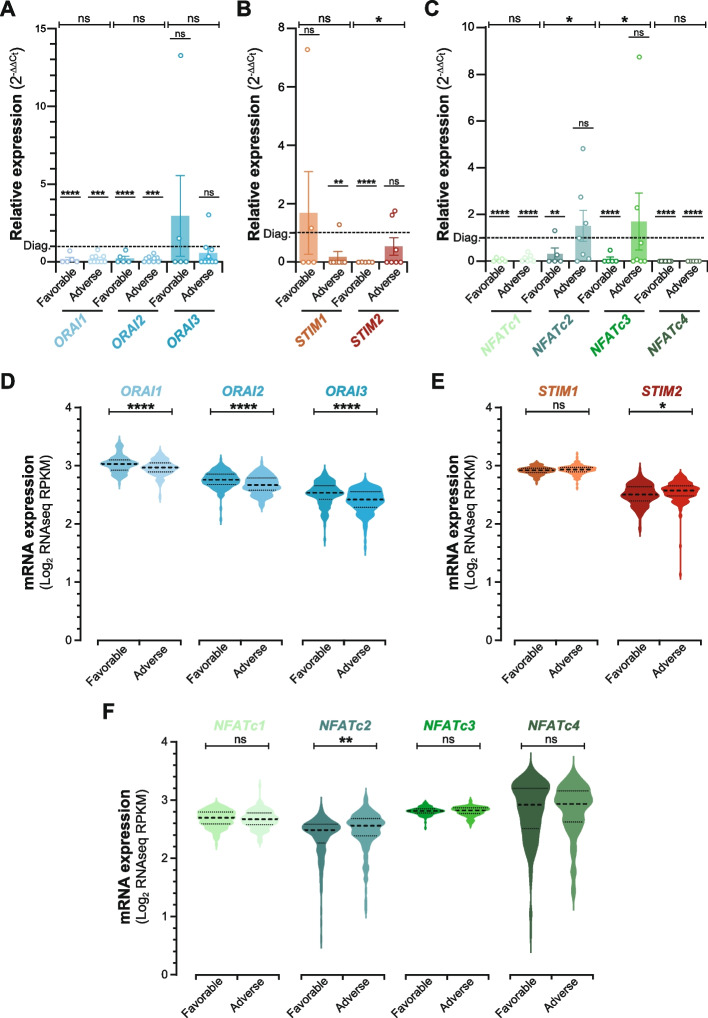
Calcium signaling gene expression stratifies AML cells and BMresLCs by prognosis. **A** Relative *ORAI1*, *ORAI2*, *ORAI3* gene expression in BMresLC compared to AML cells at diagnosis according to the clinical AML outcome (adv. *vs* fav.) and determined by RT-qPCR. *ORAI1, ORAI2*, and *ORAI3* relative gene expression was calculated by the 2^−ΔΔCt^ method. Statistical analysis was performed using Student’s *t* test (*** *p <* 0.001, ****p < 0,0001). **B** Relative *STIM1*, *STIM2* expression in BMresLC compared to AML cells at diagnosis according to the prognosis and determined by RT-qPCR. *STIM1*, *STIM2* relative expression were calculated by the 2^−ΔΔCt^ method. Statistical analysis was performed using Student’s *t* test (** *p <* 0.01, ****p < 0,0001). **C** Relative *NFATc1, NFATc2, NFATc3, NFATc4* gene expression in BMresLC compared to AML cells at the diagnosis stage according to the AML prognosis, and determined by RT-qPCR. *NFATc1, NFATc2, NFATc3,* and *NFATc4* relative gene expression was calculated by the 2^−ΔΔCt^ method. Statistical analysis was performed using Student’s *t* test (** *p <* 0.01, ****p < 0,0001). **D** Violin plots illustrating mRNA expression levels of *ORAI1, ORAI2*, and *ORAI3* in AML cells at diagnosis stratified by the adverse prognosis (*n =* 440) and favorable prognosis (*n =* 158) (public datasets, [9, 23, 24] cBioportal). Each violin represents the distribution of log₂ RPKM expression values; the central dotted marker (larger dot) indicates the median, while the upper and lower dotted markers (smaller dots) represent the interquartile range (IQR), and the violin shape reflects data density. Statistical significance between groups was assessed using the Student’s t test (****p < 0,0001). **E** Violin plots illustrating mRNA expression levels of *STIM1, STIM2* in AML cells according to the adverse (*n =* 440) and favorable prognosis (*n =* 158) (public datasets, [9, 23, 24] cBioportal). Each violin represents the distribution of log₂ RPKM expression values; the central dotted marker (larger dot) indicates the median, while the upper and lower dotted markers (smaller dots) represent the interquartile range (IQR), and the violin shape reflects data density. Statistical significance between groups was assessed using the Student’s *t* test (* *p <* 0,05). **F** Violin plots illustrating mRNA expression levels of *NFATc1, NFATc2, NFATc3*, and *NFATc4* in AML cells according to the adverse (*n =* 440) and favorable prognosis (*n =* 158) (public datasets, [9, 23, 24] cBioportal). Each violin represents the distribution of log₂ RPKM expression values; the central dotted marker (larger dot) indicates the median, while the upper and lower dotted markers (smaller dots) represent the interquartile range (IQR), and the violin shape reflects data density. Statistical significance between groups was assessed using the Student’s *t* test (** *p <* 0,01). Marks were added to distinguish between comparison to diagnosis or between adverse-risk and favorable-risk conditions

To investigate whether the calcium-NFAT axis is associated with adverse clinical outcomes in AML, we analyzed the expression of key calcium signaling genes in BMresLC by qPCR. We compared them with public RNA-seq datasets of AML cells stratified by prognosis [9, 23, 24] (Fig. 5). *ORAI1* and *ORAI2* were significantly downregulated in BMresLC relative to diagnostic AML blasts, independently of clinical risk category while *ORAI3* levels remained unchanged (Fig. 5A). BMresLC from adverse-risk patients also displayed altered expression of the ER sensors, with lower *STIM1* and higher *STIM2* expression compared with BMresLC from adverse-risk cases (Fig. 5B). qPCR profiling of *NFAT* isoforms in BMresLC (Fig. 5C) further showed that *NFATc1* and *NFATc4* remain consistently downregulated compared with diagnostic AML, with no significant differences between favorable and adverse BMresLC. In contrast, *NFATc2* expression was significantly higher in BMresLC from adverse-risk patients, while *NFATc3* was significantly lower in favorable-risk cases, indicating a risk-associated divergence in NFAT isoform usage. RNA-seq datasets from adult de novo AML [9, 23, 24] confirmed that *ORAI1*, *ORAI2*, and *ORAI3* were significantly downregulated in adverse-risk patients (Fig. 5D), and that *STIM2* was elevated, whereas *STIM1* remained unchanged (Fig. 5E). Finally, patient transcriptomic data demonstrated that *NFATc2* is significantly higher in adverse-risk AML, while *NFATc1*, *NFATc3*, and *NFATc4* do not exhibit consistent prognostic trends (Fig. 5F). Collectively, these findings indicate that although BMresLC share several calcium-related features with high-risk AML, they retain a distinct pre-therapeutic calcium-regulatory profile, clearly different from the MRD-associated calcium signature emerging after chemotherapy*.*

### BMresLC exhibit diminished store-operated calcium entry (SOCE), consistent with altered expression of calcium signaling genes

**Fig. 6:**
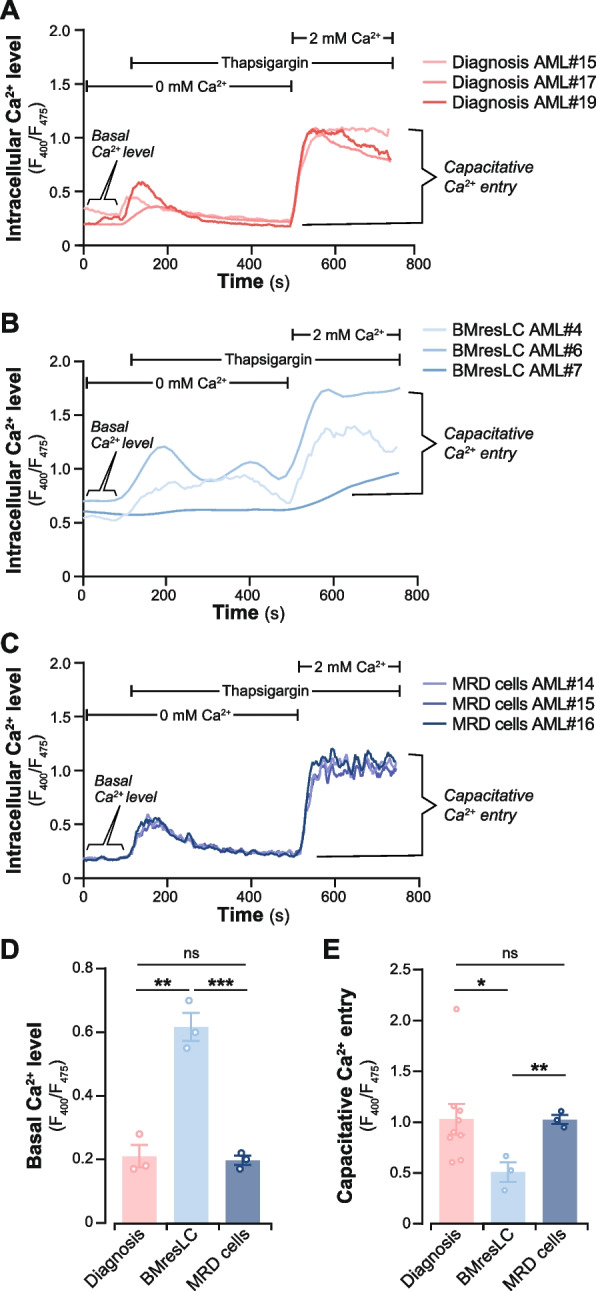
BMresLC exhibit diminished store-operated calcium entry (SOCE) in contrast with the diagnosis and MRD stage. **A** Time course of [Ca^2+^]i was analyzed by flow cytometry (Indo-AM) upon SOC activation in AML cells from samples AML#15, AML#17, and AML #19 diagnosis stage. Each point of the curve represents the mean of the signal (fluorescence intensity) from between 1000 and 10,000 cells. **B** Time course of [Ca^2+^]i was analyzed by flow cytometry (Indo-AM) upon SOC activation in BMresLC from samples BMresLC AML #6, BMresLC AML#7, and BMresLC #4. Each point represents the mean of the signal (fluorescence intensity) from between 1000 and 10,000 cells. **C** Time course of [Ca^2+^]i was analyzed by flow cytometry (Indo-AM) upon SOC activation in MRD cells from patient AML#14, AML#15, and AML#16. Each point represents the mean of the signal (fluorescence intensity) from between 1000 and 10,000 cells. **D** Ca^2+^ basal level in AML cells at diagnosis stage (pink *n =* 3), in BMresLC (light blue *n =* 3), and cells at MRD stage (dark blue *n =* 3). Statistical significance was performed using Student’s *t* test (* *p <* 0.05, ** *p <* 0.01). **e** Capacitative Ca^2+^ entry in AML cells at the diagnosis stage (pink *n =* 3), in BMresLC (light blue *n =* 3), and cells at the MDR stage (dark blue *n =* 3) was calculated according to the formula indicated in the Materials and Methods section. Statistical significance was performed using Student’s *t* test (* *p <* 0.05, ** *p <* 0.01)

To validate whether the transcriptional suppression of SOCE components translates into functional impairment, we measured intracellular calcium dynamics using the Indo-1 ratiometric probe. Following thapsigargin-induced endoplasmic reticulum calcium store depletion in calcium-free conditions, extracellular calcium was reintroduced to elicit SOCE. BMresLC displayed markedly reduced calcium influx compared to both diagnostic and MRD AML cells, as evidenced by Indo-1 fluorescence experiments (Fig. 6A, B, C). While basal intracellular calcium levels were similar in diagnostic and MRD cells, BMresLC showed significantly elevated basal calcium concentrations (Fig. 6D). The SOCE response, measured by the delta between maximal Indo-1 fluorescence ratio after calcium re-addition and the basal level, was significantly lower in BMresLC (*p <* 0.05 and *p <* 0.01 compared to diagnostic and MRD cells, respectively) (Fig. 6E). These findings confirm the functional consequences of calcium signaling gene downregulation in BMresLC, highlighting a dysregulated calcium signalling phenotype that may support long-term survival and immune evasion in the BM niche through suppressed SOCE-NFAT signaling pathways.

## Discussion

The challenge of eliminating MRD in AML lies not only in cytotoxic resistance emerging post-treatment, but also in pre-existing reservoirs within the BM niche. Using a xenotransplantation model in NSG mice, we have documented the presence of an untreated, BM-resident leukemic compartment persisting ≥ 180 days after engraftment. The spatial distribution of BMresLC observed in our 3D-cleared bone preparations suggests that these rare cells occupy canonical bone-marrow niches known to support LSC persistence and MRD-like states. Although our imaging approach does not label stromal niche components directly, the localization of BMresLC adjacent to endosteal surfaces and within perivascular regions is fully consistent with published AML studies showing that slow-cycling, drug-tolerant LSC preferentially reside in peri-endosteal niches [29, 30] and in perivascular microenvironments, including sinusoidal and arteriolar zones [31]. Importantly, chemotherapy-resistant or MRD-like AML cells have been shown to persist within these same anatomical regions [32, 33]. Our data therefore support the idea that BMresLC represent a pre-therapeutic residual population already positioned within protective LSC/MRD-supportive niches, potentially priming them for long-term survival and subsequent treatment evasion [30].

Although BMresLC share some stemness-associated transcriptional programs with LSC, they do not phenotypically correspond to a fully enriched LSC compartment. In particular, while CD34^pos^/CD38^neg^, TIM-3^pos^, and PD-L1^pos^ populations are detected, the proportion of CD34^pos^/CD38^neg^ cells in BMresLC is not significantly increased relative to diagnostic AML, indicating that BMresLC are not simply an expansion of classical LSC. Instead, BMresLC likely represent a niche-adapted, survival-oriented subfraction of LSC-like cells, shaped by BM microenvironmental cues before any therapeutic pressure, and potentially corresponding to pre-existing precursors of MRD. Interestingly, although BMresLC do not show an enrichment in the classical LSC surface phenotype (CD34^pos^/CD38^neg^), they display a clear activation of stemness-associated transcriptional programs (*KLF4, SOX2, NANOG, DNMT3B, ABCB1*). This apparent discrepancy is consistent with recent AML studies showing that LSC identity is not strictly defined by CD34/CD38 expression and that MRD cells themselves often lack enrichment in these surface markers while exhibiting strong stemness or LSC17-associated transcriptional signatures [28]. Thus, BMresLC appear to maintain a niche-conditioned, transcriptional stemness program without requiring expansion of the CD34^pos/^CD38^neg^ fraction, supporting the notion that surface marker enrichment and stemness gene expression capture different dimensions of leukemic stemness.

Functionally, BMresLC exhibit a pre-adaptive immunophenotype, characterized by the expression of surface markers TIM-3 and PD-L1, even in the absence of chemotherapy. These markers are well-established indicators of immune evasion potential and LSC-like biology [22, 34–36]. TIM-3, originally described as a functional LSC marker [22, 34–36], modulates immune evasion, whereas PD-L1 expression can be detected before therapy and has been associated with early immune escape programs in AML [37–39]. Their expression at baseline suggests that BMresLC occupy a protective, niche-supported state that may predispose them to persistence. Transcriptomic profiling reinforces this concept: BMresLC partially recapitulate MRD-associated gene-expression signatures, notably with increased *HAVCR2* (TIM-3) and *CD274* (PD-L1) expression-features typically observed in MRD. This overlap supports a continuum from pre-treatment persistent cells to therapy-selected MRD cells, rather than two unrelated populations [28]. Nevertheless, BMresLC are not identical to MRD cells. Our comparative analyses highlight distinct transcriptional programs between the two states. BMresLC upregulate early stem-like or pluripotency-associated regulators (*KLF4, SOX2, NANOG, DNMT3B, ABCB1*), whereas MRD samples show preferential enrichment for LSC17-related gene signature (*MMRN1, LAPTM4B, NYNRIN*), which are tightly associated with chemoresistance and relapse risk. These differences suggest that BMresLC reflect a pre-therapeutic, immune-evasive state, whereas MRD represents a therapy-conditioned and resistance-consolidated LSC-like phenotype.

A conceptual innovation of our study is the identification of a dysregulated, isoform-specific calcium-signaling state in BMresLC. These cells combine diminished SOCE (downregulation of *ORAI1/2* and *STIM2*), a configuration that matches the G1-restrained, slow-cycling phenotype we previously described in AML cell lines upon ORAI1 inhibition [21]. In those models, SOCE suppression induced Ki67^low^/G1 arrest, correlated with stemness and *ABCB1* expression, and promoted drug tolerance, confirming that ORAI/SOCE activity is a functional regulator of the G0-G1 balance and of persistence programs in AML. This framework aligns closely with the BMresLC phenotype, which is characterized not only by low SOCE activity and reduced *ORAI1* expression, but also by the selective suppression of *NFATc1* and *NFATc*4, while *NFATc2* and *NFATc3* remain largely unchanged. This isoform-specific pattern further supports the notion that BMresLC adopt a low-proliferative, SOCE-decoupled transcriptional state distinct from that of diagnostic AML or MRD cells. The isoform specificity of NFAT is highly relevant: NFATc1 and NFATc4 require strong SOCE-driven Ca^2^⁺ oscillations [14, 40, 41] and are associated with G1-S progression and proliferative transcription, whereas NFATc2 responds to low-amplitude, chronic Ca^2^⁺ signals and is linked to slow-cycling persistence, immune evasion, and drug tolerance [21, 41, 42]. The reduced *NFATc1/c4* expression in BMresLC therefore reflects a decoupling of SOCE-NFAT signaling from active proliferation, in line with our findings showing no accumulation of strictly quiescent (Ki67^neg^) cells, but instead a predominance of Ki67 slow-cycling cells. This pattern supports a model in which BMresLC maintain a low-proliferative, survival-oriented state shaped by the BM niche, rather than transitioning into deep, fully quiescent G0 dormancy. The ORAI isoforms also play distinct roles that shed light on our observations. ORAI1, the main SOCE pore [21, 43], is selectively suppressed in BMresLC, which may reduce Ca^2^⁺ oscillations required for NFATc1/c4 activation and G1-S transition. ORAI2, a negative regulator of SOCE amplitude [43, 44], is also downregulated. *ORAI3*, however, remains unchanged. ORAI3 is known to participate in basal Ca^2^⁺ entry and ARC channel function [45–47], supporting cell survival under stress and maintaining Ca^2^⁺ homeostasis when ORAI1-mediated SOCE is low. Its stability in BMresLC suggests that ORAI3 may help sustain basal Ca^2^⁺ levels and metabolic fitness in these slow-cycling cells. STIM isoforms further contribute to this signature: STIM2, a sensor of subtle ER Ca^2^⁺ fluctuations [44, 48–51], is reduced in BMresLC, which aligns with the observed elevation in basal Ca^2^⁺ and the attenuation of SOCE activation thresholds. Together, the ORAI1/2-STIM2 axis appears remodeled, and could thus favor persistence and protection from Ca^2^⁺ overload. Interestingly, the relative expression pattern of *ORAI* and *STIM* isoforms in BMresLC revealed a distinct and significantly altered isoform signature compared with diagnostic AML samples (Fig. S3A, B). This isoform-specific shift may contribute to explaining the unique calcium-signaling phenotype we observe.

The isoform-specific Ca^2^⁺ remodeling also explains the contrast with MRD. Post-treatment MRD cells demonstrate a partial recovery of *ORAI1/2* and, more importantly, a robust upregulation of *NFATc2*, which is strongly associated with persistence [42, 52] and immune evasion [53]. Once activated, NFATc2 drives transcriptional programs that reinforce cellular dormancy, stress tolerance, and immune-escape pathways. Notably, NFATc2 has been shown to transcriptionally induce PD-L1 expression in multiple immune and malignant contexts [53–55]. In AML, NFATc2 activation is enriched in relapsed samples, where it promotes immune-evasion circuitry and supports the survival of treatment-persistent leukemic stem/progenitor cells [41]. The co-occurrence in our data of NFATc2 upregulation and PD-L1 expression in MRD datasets is therefore consistent with a calcineurin/NFATc2-dependent immune-evasive program, tightly linked to therapy resistance and residual disease maintenance. In BMresLC, by contrast, the SOCE^low^/NFATc1-c4^low^ state reflects a pre-therapeutic, niche-conditioned equilibrium, distinct from, but potentially ancestral to the MRD phenotype. Altogether, these isoform-specific insights support a model in which BMresLC inhabit a SOCE-suppressed, basal Ca^2^⁺-high, NFATc1/c4^low^ configuration that enforces G1 restraint, limited proliferation and survival, whereas MRD cells adopt a NFATc2-dominated state shaped by chemotherapy and associated with deeper quiescence, immune escape, and chemoresistance. This mechanistic distinction helps reconcile the similarities and differences between BMresLC and MRD and underscores how Ca^2^⁺-signaling plasticity orchestrates AML persistence across disease stages.

In our study, unlike MRD-post chemotherapy cells, BMresLC demonstrated significantly increased basal Ca^2^⁺ levels despite exhibiting diminished SOCE, a paradox phenotype that may reflect compensatory calcium efflux capacity. Elevated extracellular Ca^2^⁺ concentrations in BM niches, resulting from osteoclastic resorption, create localized calcium-rich microenvironments (≥ millimolar levels) that exceed systemic serum concentration [11]. This concept has recently been confirmed by intravital imaging [56], which demonstrated strong spatial heterogeneity in extracellular Ca^2^⁺ in vivo and reported significantly elevated BM calcium levels in leukemia patients, including AML. These increases were observed both in humans and in AML mouse models [57], although whether Ca^2^⁺ returns to baseline after treatment remains unknown. Such microenvironmental Ca^2^⁺ elevations can lead to increased intracellular Ca^2^⁺ stores through passive and active Ca^2^⁺ influx, with excess calcium accumulating in the ER and mitochondria. Over time, these changes may drive adaptive remodeling of Ca^2^⁺ channels and transporters, including down-regulation of SOCE components, as a protective mechanism to prevent Ca^2^⁺ overload and apoptosis. In the context of BMresLC, the combination of high extracellular Ca^2^⁺ and reduced SOCE functionality could therefore shift the balance toward altered mitochondrial buffering, mitochondrial Ca^2^⁺ overload, or impaired OXPHOS regulation. The variability of SOCE activity observed in BMresLC, compared with the more homogeneous calcium responses measured in diagnostic and MRD cells, likely reflects the intrinsic heterogeneity of the BM niche. BMresLC reside in a microenvironment where extracellular Ca^2^⁺ levels vary spatially, and their elevated basal intracellular Ca^2^⁺ suggests local adaptation to these gradients, resulting in more dispersed SOCE responses. In contrast, MRD cells show a more uniform SOCE profile and higher *ORAI* expression, consistent with the idea that chemotherapy imposes a selective pressure that enriches for clones with a more stable, survival-optimized Ca^2^⁺/SOCE configuration. In this study, BMresLC display significantly enhanced survival compared to diagnostic AML cells under identical ex vivo conditions (Fig. S1E), indicating that they have adapted to the Ca^2^⁺-rich BM microenvironment, which sustains high basal Ca^2^⁺ but attenuates SOCE responses. Altogether, our data suggest this Ca^2^⁺-conditioned profile distinguishes BMresLC from post-treatment MRD cells and highlights potential calcium-dependent metabolic vulnerabilities for future investigation.

Beyond their biological characterization, our findings also carry potential prognostic implications. Several features identified in BMresLC mirror molecular programs classically associated with adverse-risk AML. Notably, BMresLC derived from adverse-risk patients retained higher *CD274* (PD-L1), higher *CD34* expression, features that have been repeatedly linked to immune escape, stemness, and poor outcome in AML [28, 37]. At the transcriptional level, adverse-risk AML samples were characterized by increased *NFATc2* and *NFATc3* expression, while *ORAI1-3* were markedly reduced. This “low-SOCE/high-basal Ca^2^⁺” configuration echoes the Ca^2^⁺ signature we observed in BMresLC and may reflect a pre-existing predisposition of leukemic cells from high-risk patients to adopt slow-cycling, immune-evasive survival states. Importantly, while BMresLC partially overlap with the LSC-enriched phenotype, they differ from MRD and from classical LSC transcriptional signatures. BMresLC preferentially expressed pluripotency regulators (*KLF4, SOX2, NANOG, DNMT3B, ABCB1*), whereas MRD samples and adverse-risk diagnostic samples displayed higher levels of LSC17 genes, such as *MMRN1*, *LAPTM4B,* and *NYNRIN*, a signature strongly predictive of relapse and survival [28]. This divergence suggests that BMresLC may represent a pre-therapeutic, niche-adapted persistent reservoir, whereas MRD and adverse-risk AML reflect a more consolidated, therapy-conditioned LSC program. Taken together, these results indicate that calcium-signaling remodelling, particularly *NFATc2* activation and *ORAI* downregulation, may contribute to high-risk AML biology. The observation that several of these adverse-risk features are already detectable in BMresLC before treatment raises the possibility that early Ca^2^⁺/NFAT profiles could serve as pre-MRD prognostic indicators, identifying patients at risk of chemoresistance or relapse even before therapy begins.

Our model raises critical questions regarding clonality and subclone origin: Are BMresLC derived from pre-existing LSC subclones, or do they arise through selective niche adaptation? Single-cell lineage tracing and bulk & mutational profiling will provide crucial answers [58]. Regarding therapy-induced reprogramming: Does chemotherapy provoke transition from the calcium-dysregulated to calcium-reactive MRD state? Longitudinal xenograft studies are needed to map these shifts dynamically [52]. The unique calcium phenotype renders BMresLC potentially vulnerable to agents that further impede SOCE, triggering differentiation or sensitization to chemotherapy. This strategy echoes emerging therapeutic paradigms targeting niche-dependent LSC physiology [59]. Finally, the immune-evasive profile suggests that early application of checkpoint inhibitors, perhaps in concert with calcium modulators, could be a potent strategy to eradicate pre-MRD.

Our study presents certain limitations that should be acknowledged, largely inherent to the PDX model and the rarity of BMresLC. Although NSG mice lack adaptive immune components, making it difficult to fully recapitulate immune-mediated interactions, this model remains one of the most robust systems for preserving primary human AML biology and enabling the detection of rare, treatment-naïve BM-resident leukemic cells. As a result, immune-evasive programs observed in BMresLC must be interpreted in the context of a partially immunodeficient niche, but their consistent detection across mice suggests that they reflect genuine, pre-therapeutic properties rather than artifacts of the system. A second consideration relates to the extremely small number of BMresLC that limits the extent of functional perturbation assays that can be performed ex vivo. Nevertheless, we were able to obtain reliable Ca^2^⁺ kinetics, SOCE measurements, and cell-cycle profiles, which provide strong mechanistic support for the Ca^2^⁺-dependent adaptations identified in these cells.

**Fig. 7:**
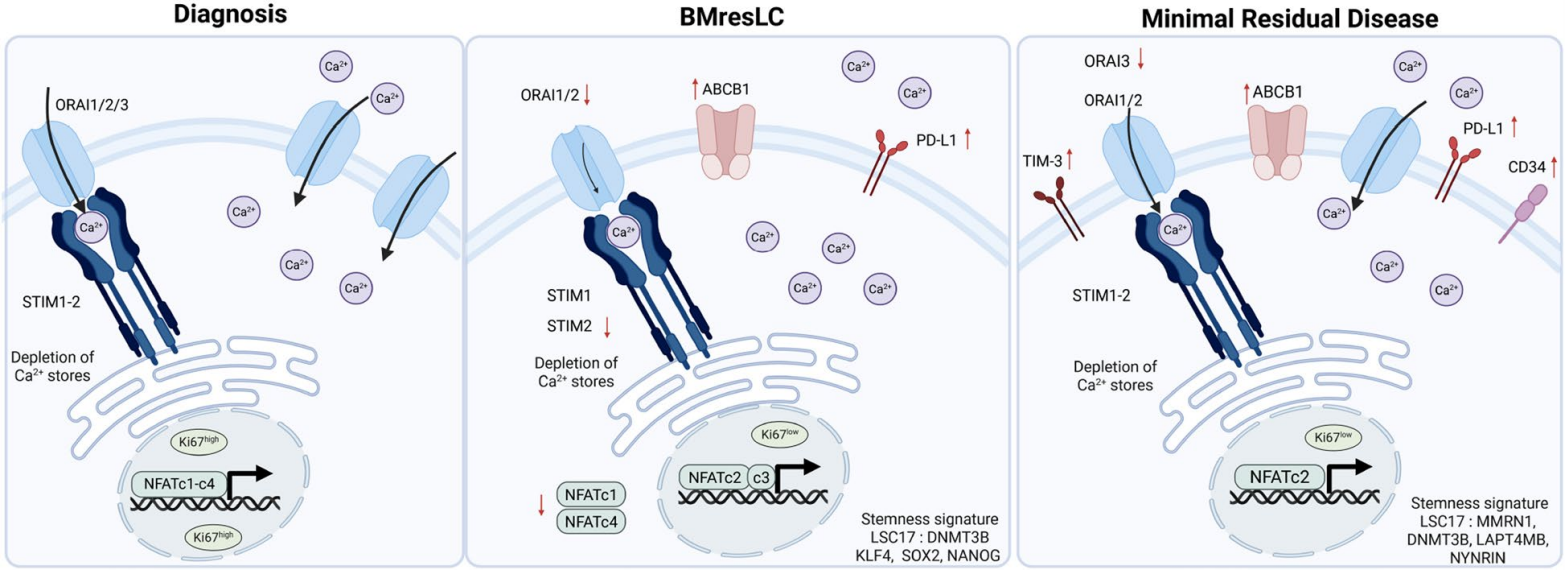
Schematic overview of calcium-signaling states, stemness programs, and proliferative features across diagnostic AML cells, BMresLC, and MRD cells. **A** At diagnosis, AML cells display elevated SOCE activity, driven by ORAI1-2 and STIM1-2, resulting in strong Ca^2^⁺ influx and activation of NFATc1-NFATc4 transcription factors. This stage is associated with a high Ki67 proliferation status. **B** In BMresLC, SOCE is markedly diminished due to downregulation of *ORAI1, ORAI2*, and *STIM2*. These cells show elevated basal Ca^2^⁺ levels, repression of *NFATc1* and *NFATc4*, and induction of stemness and pluripotency-associated factors (*KLF4, SOX2, NANOG*), along with immune-evasive markers (PD-L1). BMresLC contain a small but detectable Ki67^neg^ quiescent subpopulation and show a clear enrichment in Ki67^low^ slow-cycling cells, reflecting a predominantly low-proliferative phenotype. **C** In MRD cells, cancer cells regain high SOCE activity with ORAI1-2 expression recovering and exhibit reduced expression of *ORAI3*, and increased expression of *NFATc2*. These cells also show enhanced expression of immune-evasive markers (*TIM-3, PD-L1*), drug-resistance transporters (*ABCB1*), and stemness-related genes, including LSC17 stemness genes (*MMRN1*, *LAPTM4B*, *DNMT3B, NYNRIN*) and a deeper quiescence (Ki67^neg^) and low-proliferation status (Ki67.^low^)

In conclusion, despite its limitations, our study identifies a rare, treatment-naïve, BM-resident leukemic cell state characterized by quiescence, slow cycling, stemness, immune evasion, pluripotency gene expression, and a distinct “calcium signaling-dysregulated” phenotype (Fig. 7). These cells likely underpin MRD, even before therapeutic exposure. Consequently, integrating niche-disrupting, immune checkpoint, and calcium-targeted strategies may be crucial for eliminating these pre-MRD reservoirs and achieving curative remission.

## Materials and methods

### BM AML patient samples at diagnosis and during MRD

BM samples of AML patients were obtained at diagnosis with > 80% of leukemic cells and during the MRD stage of the disease (less than 5% of leukemic cells). These patient samples were collected in the department « Maladies du Sang» of Claude Huriez Lille Hospital. Clinical information for all AML patients, including age, prognostic classification, and baseline characteristics, is provided in Table S1. A density gradient separation using Pancoll solution (PAN-Biotech, Aidenbach, Germany), was performed to isolate peripheral blood mononuclear cells (PBMCs). Red blood cells were lysed, and lymphocytes were depleted using immunomagnetic negative selection (StemCell, CD3 and CD20).

### Patient-derived xenografts to collect/isolate the rare BM-resident/tolerant leukemic cells

The immunodeficient NOD/SCID/IL2Rγc^null^ (NSG) mouse model was used for engraftment of malignant human hematopoietic cells as previously described [22]. 5.10^6^ AML blast cells (isolated at diagnosis) or PBS (negative control mice) were injected into the tail vein of 10 unconditioned NSG mice. To control blood leukemic cell dissemination, 60 µL of blood was collected every two weeks by the submandibular method, followed by flow cytometry analysis of the sample (anti-human CD45-FITC *vs* anti-mouse CD45-APC). Around 40–60% of mice, according to the AML patient sample, were positive for engraftment, i.e., > 5% of leukemic cells in peripheral blood, while the remaining ones displayed no peripheral blood leukemic cell dissemination or < 1% of leukemic cells, for a period post-AML cell injection between 180 and 250 days. When continuous negative AML cell blood detection or a constant percent less than 1% was monitored after a minimum of 180 days post-injection, mice were euthanized by cervical dislocation following international guidelines. Murine BM cells, including or not, BM resident human leukemic cells, were collected by flushing tibias and femurs with PBS and frozen for future analysis.

### Detection of rare BM-resident/tolerant human leukemic cells in NSG mice

The presence of BM resident leukemic cells was detected by flow cytometry and qRT-PCR by using human-specific cell surface markers (B2M, CD33, CD34, CD38, TIM-3, PD-L1). Murine BM cells isolated from control non-injected mice were used as negative control for qRT-PCR and flow cytometry experiments, and no positive signal was observed with all human Taqman probes and anti-human cell surface markers.

### Immunophenotyping of AML cells

AML cells isolated from BM samples at diagnosis and during MRD, and BM-resident AML leukemic cells (in murine BM cells) were stained with fluorochrome-conjugated antibodies (all from Biolegend, San Diego, CA, USA) for 30 min at room temperature (RT) in PBS with 10% SVF. Antibodies were used at a 1:100 dilution (except for the PE-Cy7-conjugated CD33 antibody, used at a 1.25:1000 dilution), and matching isotype antibodies were used at the same final concentration. Then, the cells were rinsed and resuspended in PBS before being analyzed by flow cytometry. Compensation beads (Invitrogen) were used to establish a matrix of compensation. Gating was determined based on negative control cell staining with corresponding isotype antibodies. If combined with the calcium influx and cell viability assays, APC-conjugated CD34, PE-conjugated TIM3, PE-Cy7-conjugated CD33, and Brilliant Violet 421 (BV421)-conjugated CD38 antibodies, PerCP-Cyanine5.5-conjugated PD-L1, and PE-conjugated Beta-2-microglobuline (Biolegend) were used. Cells were loaded with a DyeTM 750/777 Fixable Viability Staining Kit for 30 min at RT and protected from light to assess their viability.

### Calcium influx assay by flow cytometry

SOCE activity in AML cells at diagnosis, during MRD, and rare NSG BM-resident human xenograft leukemic cells was performed as previously described [22]. Briefly, cells were loaded with the ratiometric dye indo-1-AM (Thermo Fisher Scientific, Waltham, MA, USA) as a Ca^2+^ indicator. One million cells per milliliter were loaded with 0.7 μM indo-1-AM in the corresponding medium for 30 min at 37 °C in the dark and then washed. Cells of interest were gated according to their expression of human cell surface markers described in the above section. The evolution of the intracellular calcium concentration ([Ca^2+^]i) was measured every 5 s as the F400 nm/F475 nm (RF400/F475) ratio of fluorescence with a flow cytometer UV light. Baseline [Ca^2+^]i was acquired in a 0 mM Ca^2+^ solution containing 140 mM NaCl, 5 mM KCl, 1 mM MgCl2, 5 mM glucose, and 10 mM HEPES (pH 7.4). After 120 s, the cells were treated with 1 µM of the sarco/endoplasmic reticulum calcium ATPase (SERCA) inhibitor thapsigargin (Focus Biomolecules). After 420 s of treatment with thapsigargin, a Ca^2+^-containing solution was added to the cells (final [Ca^2+^]_e_ 2 mM). Fluorescence was monitored with an LSR-Fortessa X20 flow cytometer, and median fluorescence values (*n =* 1000–10,000 cells for each time point) were extracted for further analysis. The capacitative calcium entry (also noted as SOCE for store-operated calcium entry) (ΔiRF400/F475) was calculated as previously described [21]. Graphs were plotted using GraphPad Prism software.

### Cell cycle assay

To assess the cycling status (Ki67 negative quiescent or positive cycling cells) of human leukemic cells at diagnosis or within mice BM, the Ki67 protein expression level was analyzed by flow cytometry. Cells were first fixed and permeabilized with 70% ethanol for 30 min at −20 °C. Cells were next rinsed and stained with the following anti-human antibodies: CD34-APC; CD38-BV421; PD-L1-PerCP-Cy5.5, TIM-3-PE at a dilution of 1:100, and CD33-PeCy7 at a 1.25:2000 (Biolegend) and KI67-FITC (Abcam) at 1:100 dilution for 30 min at 4 °C. Then, the cells were washed and resuspended in 200 µL PBS until flow cytometry analysis.

### RNA extraction, qRT-PCR

Total cellular RNA was extracted following the manufacturer’s protocol (Qiagen RNeasy Mini Kit). Then, RNA was transcribed into cDNA using random hexamers and the *High Capacity Reverse Transcription kit* from Applied Biosystems (Foster City, CA, USA). All qRT-PCRs were performed using specific human TaqMan fluorescent probes (human *ORAI1-3, STIM1-2, NFAT1-4, MMRN1, LAPTM4B, NYNRIN, KLF4, SOX2, NANOG, DNMT3B, ABCB1* and *BETA-2 MICROGLOBULIN* or *B2M*) provided by Applied Biosystems, Bedford, MA, USA. Duplex qPCR was performed, and discrimination of the genes of interest from the reference gene was allowed owing to 2 distinct fluorophore dyes, FAM and VIC probes for the genes of interest and the reference gene, respectively. The relative expression ratio for each gene was calculated by the 2^−ΔΔCt^ method. The calculated values represent the expression level for each gene relative to the expression of *B2M*, which was used as the endogenous control. No expression denotes no detection in 10 ng of cDNA after 60 cycles of amplification.

### In situ or in toto immunostaining of human resident leukemic cells in NSG mice BM and BM 3DISCO clearing

Mice bones (tibia and femur) were collected as described in the section above. Bones were fixed overnight in PFA at 4 °C. Decalcification was performed by 48 h incubation with Osteosoft solution (Sigma). Samples were successively incubated with PBSNaGT (PBS 1X, gelatin (0.2%), Triton X100 (0.5%), Na Azide (0.01%) for permeabilization and then with PBSNaGT 5% DMSO at 37 °C for 4 days under agitation. Bone tissues were next blocked using Goat serum at 3% and incubated with anti-human B2M (Abcam) and anti-human Ki67 (Abcam) antibodies at 37 °C for 7 days, protected from light. Samples were next washed 3 times during 30 min with PBDNaGT and in PBSNaGT overnight at 4 °C. For tissue clearing, the 3DISCO modified protocol was followed as previously described [60]. Samples were dehydrated in 50% THF (tetrahydrofuran) (Sigma 186,562-2L) at RT overnight under agitation, at 80% in THF for 1 h, and in 100% THF (2 × 90 min). Bones were washed in DCM (Dichlorométhane) (Sigma) for 30 min at RT and then in DBE (Dibenzylether) (Sigma) for 2 h at RT and washed with DBE, and were stored in individual light-absorbing glass vials (Rotilabo, Roth) at RT. In these conditions, samples could be stored and imaged for up to 9 months without any significant fluorescence loss.

### 3D imaging of BM and image processing

3D imaging was performed with an ultramicroscope I (LaVision BioTec) using ImspectorPro software (LaVision BioTec). The light sheet was generated by a laser (wavelength 488 and 561 nm, Coherent Sapphire Laser and 640 nm, Coherent OBIS 640-100LX laser, LaVision BioTec). A binocular stereomicroscope (MXV10, Olympus) with a 2 × objective (MVPLAPO, Olympus) was used at different magnifications (0.8 ×, 1 ×, 1.25 ×, 1.6 ×, 2 ×, 2.5 ×, 3.2 ×, 4 ×, 5 ×, and 6.3 ×). Samples were placed in an imaging reservoir made of 100% quartz (LaVision BioTec) filled with DBE and illuminated from the side by the laser light. Images were acquired with an Andor Neo SCMOS camera (Bitplane). The stepsize between each image was fixed to 3 μm. Image processing was performed using Imaris × 64 software version 9.1.

### Analysis of public datasets

RNA-seq datasets of human AML samples at diagnosis, at MRD/residual, at relapse, and according to the prognosis (ELN 2022), adverse and favorable, are available in the public domain (https://www.cbioportal.org, accessed on 15 March 2022) and were generated previously [9, 23, 24]. The RNA-seq expression unit used was log₂ RPKM. Raw data were extracted and analyzed as previously described [25].

### Statistical analysis

The number of replicates and the statistical tests that were used for each experiment are specified in the relative figure legend. For statistical analysis, GraphPad Prism software was used. Data was considered statistically significant for *p <* 0.05.

## Supplementary Information

Supplementary Material 1: Table S1. ELN 2022 Classification and clinical/biological characterization of AML patients.

Supplementary Material 2: Figure S1. Identification and phenotypic characterization of rare BMresLC in xenografted mouse BM. (A) Heatmap showing, among total murine BM cells, the frequency of human BMresLC expressing the indicated surface markers. Human B2M^pos^ cells are shown in pink, B2M^pos^/Ki67^pos^ cells in green, and CD33^pos^ myeloid leukemic cells in blue. Samples were annotated by patient identifier (AML#) and by mouse replicate (letter), such that each xenografted mouse derived from the same patient sample is uniquely labeled (B) Representative flow cytometry plots illustrating the gating strategy for BMresLC (Red A780-60^neg^ viable cells) and confirming human myeloid identity through hCD33 expression, compared with diagnostic AML blasts. (C) Representative flow cytometry plots showing Ki67 expression within BMresLC gated on hCD33^pos^ cells. (D) Quantification of Ki67^low^ slow-cycling cells in diagnostic AML samples compared with BMresLC, highlighting the slow-cycling phenotype of residual BM populations. Statistical significance was performed using Student’s t test (** *p* < 0.01). (E) Percentage of viable cells (Red A780-60^neg^) in diagnostic AML samples *versus* BMresLC, demonstrating the enhanced survival capacity of BM-resident leukemic cells under identical *ex vivo* conditions. Statistical significance was performed using Student’s t test (** *p* < 0.01).

Supplementary Material 3: Figure S2. Flow-cytometric analysis of CD34^pos^/CD38^neg^, PD-L1^pos^, and TIM-3^pos^ BMresLC subpopulations (A) Heatmap showing the proportion of BMresLC among BM cells from several mice, and expressing the following surface markers analyzed by flow cytometry: human CD34^pos^CD38^neg^ cells (pink gradient), PD-L1^pos^ cells (red gradient), and TIM-3^pos^ cells (dark red gradient). Samples were annotated by patient identifier (AML#) and by mouse replicate (letter), such that each xenografted mouse derived from the same patient sample is uniquely labeled (B) Flow cytometry-based quantification of human CD34^pos^/CD38^neg^ cells (pink dot), PD-L1^pos^ cells (red triangle), and TIM-3^pos^ cells (dark red square) in patient samples at diagnosis and among hBMresLC from xenografted mouse BM. Statistical significance was performed using Student’s t test (** *p*< 0.01).

Supplementary Material 4: Figure S3. Expression of SOCE pathway actors shows isoform-dependent variation according to disease stage. (A) Relative *ORAI1, ORAI2, *and* ORAI3* gene expression in BMresLC and AML cells at the diagnosis stage was determined by RT-qPCR. *ORAI1, ORAI2*, and *ORAI3* relative gene expression was calculated by the 2^-ΔΔCt^ method and normalized with the isoform *ORAI1* value (diagnosis). Statistical significance was performed using Student’s *t* test (* *p* < 0.05). (B) Relative *STIM1, STIM2* gene expression in BMresLC and AML cells at the diagnosis stage was determined by RT-qPCR. *STIM1, STIM2* relative gene expression were calculated by the 2^-ΔΔCt^ method, normalized with the isoform *STIM1* value (diagnosis). Statistical significance was performed using Student’s *t* test (* *p* < 0.05). (C) Relative *NFATc1, NFATc2, NFATc3, *and *NFATc4 *expression in BMresLC and AML cells at diagnosis determined by RT-qPCR. *NFATc1, NFATc2, NFATc3*, and *NFATc4* relative expression was calculated by the 2^-ΔΔCt^ method and normalized with the isoform *NFATc1* value (diagnosis). Statistical significance was performed using Student’s t test (* *p* < 0.05, *** *p* < 0.001).

## Data Availability

All data that support the results of this study are contained in the main article and its supplementary information files. The corresponding author can provide access to the raw data upon a reasonable inquiry.

## Abbreviations

3DISCO: 3D imaging of solvent-cleared organs
AML: Acute Myeloid Leukemia
BM: Bone Marrow
B2M: Beta-2 microglobulin
BMresLC: Bone marrow resident leukemic cells
ER: Endoplasmic reticulum
HSC: Hematopoietic Stem Cell
LSC: Leukemic Stem Cell
MRD: Minimal Residual Disease
NFAT: Nuclear Factor of Activated T cell
NSG: NOD/SCID/IL2Rγ^null^ mice
OXPHOS: Oxidative Phosphorylation
PD-L1: Programme death ligand 1
PDX: Patient-Derived Xenograft
qPCR: Quantitative Polymerase Chain Reaction
RPKM: Reads Per Kilobase per Million mapped reads
SC: Stem-cell
SOCE: Store-Operated Calcium Entry
STIM: Stromal Interaction Molecule
