# Supplementary figures and images for: Pre-therapeutic bone marrow-resident leukemic cells in acute myeloid leukemia exhibit a distinct dysregulated calcium signature and stem-like profile reflecting minimal residual disease precursors
Source: J Exp Clin Cancer Res. 2026 Jan 9;45:55. doi: 10.1186/s13046-025-03634-x (PMC12911078; doi:10.1186/s13046-025-03634-x)

**A**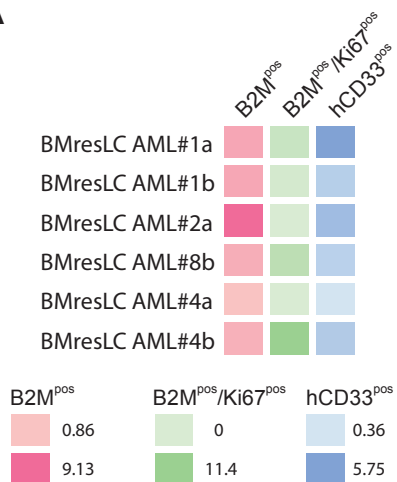**B**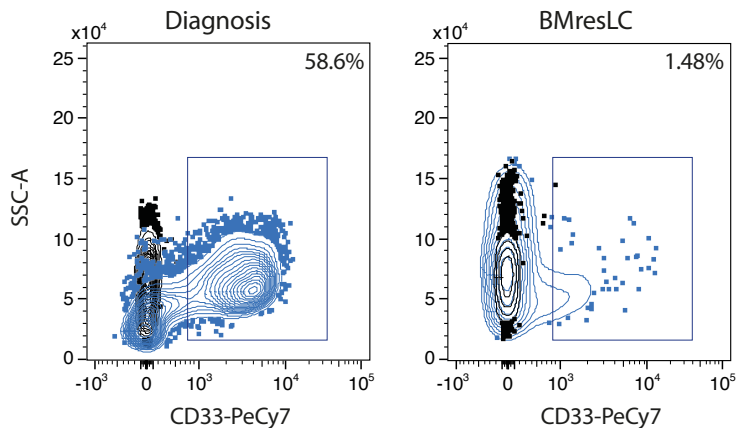**C**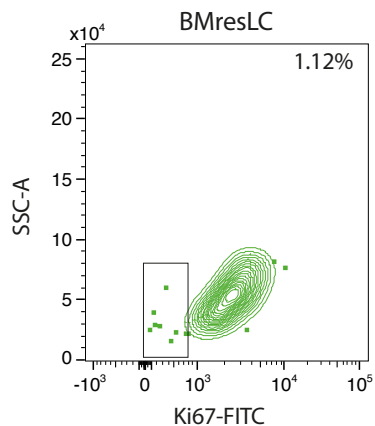**D**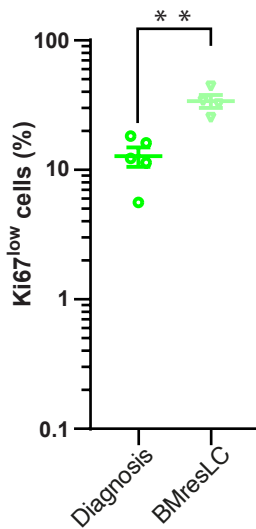**E**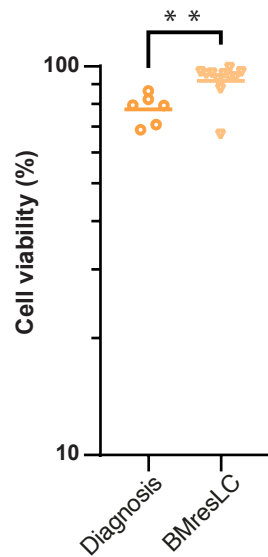

Supplement: Supplementary file 2 — Supplementary Material 2: Figure S1. Identification and phenotypic characterization of rare BMresLC in xenografted mouse BM. (A) Heatmap showing, among total murine BM cells, the frequency of human BMresLC expressing the indicated surface markers. Human B2Mpos cells are shown in pink, B2Mpos/Ki67pos cells in green, and CD33pos myeloid leukemic cells in blue. Samples were annotated by patient identifier (AML#) and by mouse replicate (letter), such that each xenografted mouse derived from the same patient sample is uniquely labeled (B) Representative flow cytometry plots illustrating the gating strategy for BMresLC (Red A780-60neg viable cells) and confirming human myeloid identity through hCD33 expression, compared with diagnostic AML blasts. (C) Representative flow cytometry plots showing Ki67 expression within BMresLC gated on hCD33pos cells. (D) Quantification of Ki67low slow-cycling cells in diagnostic AML samples compared with BMresLC, highlighting the slow-cycling phenotype of residual BM populations. Statistical significance was performed using Student’s t test (** p < 0.01). (E) Percentage of viable cells (Red A780-60neg) in diagnostic AML samples versus BMresLC, demonstrating the enhanced survival capacity of BM-resident leukemic cells under identical ex vivo conditions. Statistical significance was performed using Student’s t test (** p < 0.01). [file 13046_2025_3634_MOESM2_ESM.pdf]

**A**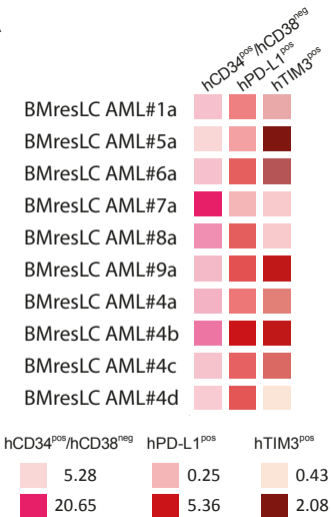**B**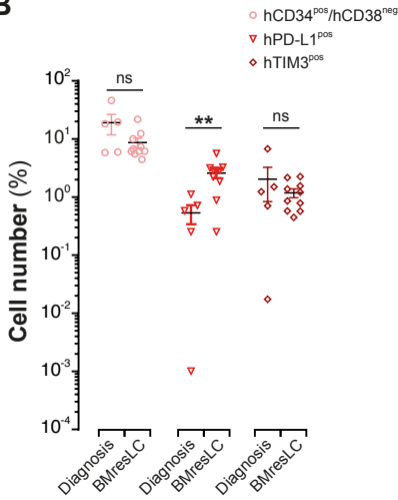

Supplement: Supplementary file 3 — Supplementary Material 3: Figure S2. Flow-cytometric analysis of CD34pos/CD38neg, PD-L1pos, and TIM-3pos BMresLC subpopulations (A) Heatmap showing the proportion of BMresLC among BM cells from several mice, and expressing the following surface markers analyzed by flow cytometry: human CD34posCD38neg cells (pink gradient), PD-L1pos cells (red gradient), and TIM-3pos cells (dark red gradient). Samples were annotated by patient identifier (AML#) and by mouse replicate (letter), such that each xenografted mouse derived from the same patient sample is uniquely labeled (B) Flow cytometry-based quantification of human CD34pos/CD38neg cells (pink dot), PD-L1pos cells (red triangle), and TIM-3pos cells (dark red square) in patient samples at diagnosis and among hBMresLC from xenografted mouse BM. Statistical significance was performed using Student’s t test (** p< 0.01). [file 13046_2025_3634_MOESM3_ESM.pdf]

A

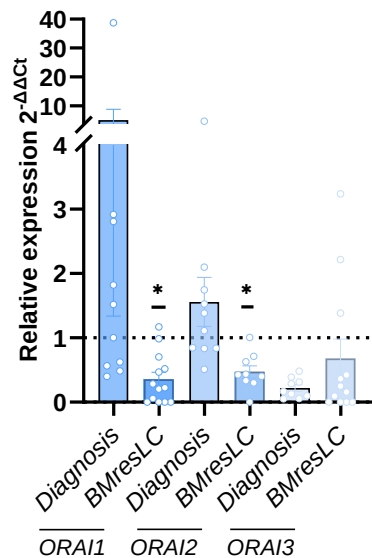

B

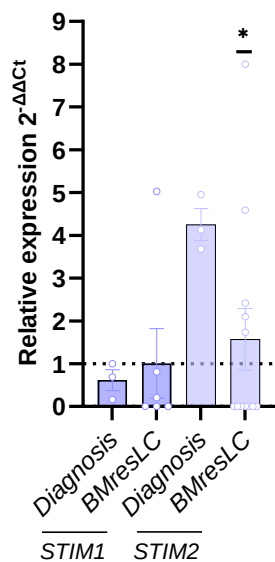

C

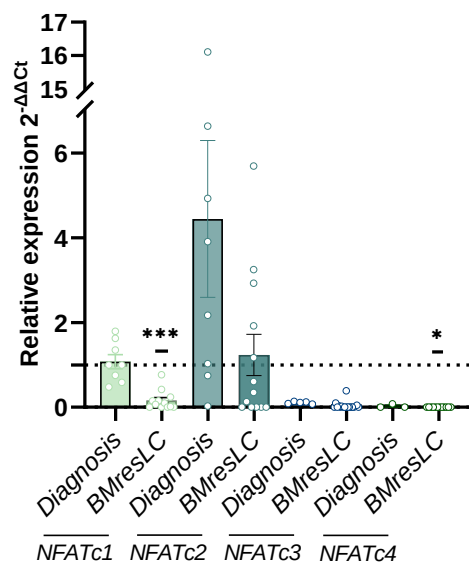

Supplement: Supplementary file 4 — Supplementary Material 4: Figure S3. Expression of SOCE pathway actors shows isoform-dependent variation according to disease stage. (A) Relative ORAI1, ORAI2, and ORAI3 gene expression in BMresLC and AML cells at the diagnosis stage was determined by RT-qPCR. ORAI1, ORAI2, and ORAI3 relative gene expression was calculated by the 2-ΔΔCt method and normalized with the isoform ORAI1 value (diagnosis). Statistical significance was performed using Student’s t test (* p < 0.05). (B) Relative STIM1, STIM2 gene expression in BMresLC and AML cells at the diagnosis stage was determined by RT-qPCR. STIM1, STIM2 relative gene expression were calculated by the 2-ΔΔCt method, normalized with the isoform STIM1 value (diagnosis). Statistical significance was performed using Student’s t test (* p < 0.05). (C) Relative NFATc1, NFATc2, NFATc3, and NFATc4 expression in BMresLC and AML cells at diagnosis determined by RT-qPCR. NFATc1, NFATc2, NFATc3, and NFATc4 relative expression was calculated by the 2-ΔΔCt method and normalized with the isoform NFATc1 value (diagnosis). Statistical significance was performed using Student’s t test (* p < 0.05, *** p < 0.001). [file 13046_2025_3634_MOESM4_ESM.pdf]
